# Mutations of DnaA-boxes in the *oriR* region increase replication frequency of the MiniR1–1 plasmid

**DOI:** 10.1186/s12866-018-1162-3

**Published:** 2018-04-03

**Authors:** Yuan Yao, Sukhbold Enkhtsetseg, Ingvild Odsbu, Lifei Fan, Morigen Morigen

**Affiliations:** 1 State Key Laboratory of Reproductive Regulation & Breeding of Grassland Livestock,School of Life Sciences, Inner Mongolia University, Hohhot, 010070 China; 2 Department of Public Health Sciences, Karolinska Institutet, Stockholm, Sweden

**Keywords:** MiniR1–1 replication, DnaA-boxes, Complete genome sequence, *E. coli* Cell cycle

## Abstract

**Background:**

The MiniR1–1 plasmid is a derivative of the R1 plasmid, a low copy cloning vector.

**Results:**

Nucleotide sequencing analysis shows that the MiniR1–1 plasmid is a 6316 bp circular double-stranded DNA molecule with an *oriR*1 (origin for replication). The plasmid carries the *repA*, *tap*, *copA* and *bla* genes, and genes for ORF1 and ORF2. MiniR1–1 contains eight DnaA-binding sites (DnaA-boxes). DnaA-box1 is in the *oriR*1 region and fully matched to the DnaA-box consensus sequence, and DnaA-box8, with one mismatch, is close to the *copA* gene. The presence of the MiniR1–1 plasmid leads to an accumulation of the D-period cells and an increase in cell size of slowly growing *Escherichia coli* cells, suggesting that the presence of MiniR1–1 delays cell division. Mutations in the MiniR1–1 DnaA-box1 and DnaA-box8 significantly increase the copy number of the plasmid and the mutations in DnaA-box1 also affect cell size. It is likely that titration of DnaA to DnaA-boxes negatively controls replication of the MiniR1–1 plasmid and delays cell division. Interestingly, DnaA weakly interacts with the initiator protein RepA in vivo.

**Conclusion:**

DnaA regulates the copy number of MiniR1–1 as a negative factor through interacting with the RepA protein.

**Electronic supplementary material:**

The online version of this article (10.1186/s12866-018-1162-3) contains supplementary material, which is available to authorized users.

## Background

The R1 plasmid is a large conjugative plasmid of size 95.8 kb [1, 2]. It is a low copy number plasmid and belongs to the IncFII group [3]. The plasmid carries *bla* (ampicillin), *cat* (chloramphenicol), *neo* (kanamycin), *aad* (streptomycin/spectinomycin) and *sul* (sulfonamide) genes in the R-determinant, which contains three insertion sequences and a Tn4 transposon [4]. The basic replicon elements and stability systems including partition (*parA*), killing (*hok*/*sok* or *parB*), second killing (*parD*), and conjugation (*tra*) gene cassettes are distributed around the plasmids [4]. ParA is responsible for partition of the plasmid, ensuring that each daughter cell receives certain copies of the plasmid [4]. The *hok* gene product kills cells which have not received the plasmid at cell division [5]. Proteins encoded by the *tra* operon mediate conjugal transfer of the plasmid into plasmid-free cells [6]. The R1 basic replicon element is about 2 kb, and contains *oriR*1, and *repA, copA, copB* and *tap* genes [7]. Products of these genes are required for initiation of plasmid replication and copy number control. The *copA* gene encodes an antisense RNA that limits translation of RepA protein. The other gene, *tap*, encodes a small leader peptide (Tap, translational activator peptide) [8].

The R1 plasmid replication initiates at *oriR*, and proceeds unidirectionally according to the Theta mode [2, 9]. Initiation of replication depends on the plasmid RepA and the host DnaA proteins [10]. DnaA recognizes and binds specifically to a DnaA-box with a conservative 9-mer TTA/TTNCACA sequence in *oriR* [11] only when RepA is bound to the sequence immediately downstream of the DnaA-box [10]. RepA triggers initiation of replication of the R1 plasmid efficiently when DnaA is bound to the DnaA-box in *oriR* [10, 12]. However, RepA opens the double-helix and facilitates assembly of the replisome at *oriR*, and can support replication of the R1 plasmid in vivo in the absence of DnaA [13, 14]. Controversially, it is reported that replication of the R1 plasmid is inefficient in the absence of DnaA and presence of normal levels of RepA [15].

Several MiniR1 plasmids have been reported. The Rsc11 plasmid is a derivative of R1*drd*-19B2 [16], containing a transposon carrying the *bla* gene and the basic replicon region. The pKN177 and pKN182 plasmids are originated from the R1 copy mutant pKN104 [2]. The pJEL109 plasmid is another R1 derived vector with 1–2 copies per host chromosome [17], carrying the R1 origin of replication, the *bla* gene from Tn3 and unique cloning sites [18]. In this work, pJEL109 was renamed as MiniR1–1 and its complete genome was sequenced. Further, we found that the presence of the MiniR1–1 plasmid delayed cell division, and affected initiation of chromosome replication but not chromosome segregation. Mutagenesis analysis showed that mutations in the MiniR1–1 DnaA-box1 and DnaA-box8 increased copy number of the plasmid and affected both cell size and growth rate.

## Methods

### Bacterial strains and plasmids

All bacterial strains used were *E. coli* K-12 and are listed in Table 1.

**Table 1:** Strains and plasmids

Strain or plasmid	Genotype	Reference
MG1655	Wild-type	[36]
MOR70	MG1655/MiniR1–1	[31]
MOR72	MG1655/pACYC177	[31]
MiniR1–1	*oriR1, bla, repA, tap, copA*	This work
BTH101	F *cya-99 araD139 galE15 galK16 rpsL1* (Str^r^) *hsdR2 mcrA1 mcrB1*	[35]
pKNT25	repp15AKmR placT25(pSU40 derivative)	[37]
pUT18	repColE1ApR placT18(pUC19 derivative)	[37]
pKNT-*dnaA*	*dnaA* fused to T25 on pKNT25	This work
pUT*-repA*	*repA* fused to T18 on pUT18	This work
pMOR1	MiniR1–1-*datA*	[31]

### Growth conditions and growth rate determination

Cells were grown exponentially at 30 °C in AB minimal medium [19] supplemented with 10 μg/ml thiamine, 0.2% Ala, 20 μg/ml Met, 20 μg/ml Trp and 20 μg/ml His (ABT) [20], or AB supplemented with 10 μg/ml thiamine, 0.2% glucose and 0.5% casamino acids (ABTG-CAA) [21]. 50 μg/ml of ampicillin was added when required for selection. To determine the doubling time of an exponentially growing culture, the OD_450_ of the culture was measured at different time points. Using the values of log_2_OD as the Y-axis and the time (T_X_-T_0_, T_0_ represents the time at which the first OD value was measured; T_X_ represents the time at which the OD values were measured) as the X-axis, the doubling time (minutes) could be calculated from the formula y = ax+b, where ‘a’ (slope) represents the doubling time (minutes) of the cell culture analyzed.

### Flow cytometry

Exponentially growing cells in poor medium were centrifuged for collection, or treated with two drugs (300 μg/ml rifampicin and 10 μg/ml cephalexin) to inhibit both initiation of replication and cell division for several generations as described [20]. Functions of rifampicin and cephalexin are as mentioned previously [22, 23]. Cells untreated or treated with the drugs were fixed in 70% ethanol and then analyzed by flow cytometry (BD LSRFortessa) after one wash in Tris-HCl buffer (pH 7.5) and subsequent stainning with Hoechst 33,258 (1.5 μg/ml) for 30 min. The percentage of B-period cells was determined from the amount of one-chromosome cells estimated by flow cytometry; the percentage of D-period cells was calculated from the amount of two-chromosome cells; the percentage of C-period cells was determined from the amount of cells with DNA contents between the one-chromosome and two-chromosomes (Additional file 1: Figure S1). Preparation of standard sample and post analysis were as described previously [20].

### Genome sequencing of the MiniR1–1 plasmid

The MiniR1–1 plasmid genome was sequenced using primers targeting the *datA* sequence in pMOR1, a derivate of MiniR1–1, carrying a *datA* site inserted between *Bam*HI and *Hin*dIII sites [24]. Subsequently using the sequences links at the *Bam*HI and *Hin*dIII sites, the genome sequence of MiniR1–1 was determined.

### Mutagenesis

Site directed mutagenesis kit was used to introduce one or more mutations into DnaA-boxes or ORFs of the MiniR1–1 plasmid. Using the partial overlapping primer design technique [25], primers were designed as listed in Table 2. The mutated plasmids, positions of mutations introduced, and sequences before and after mutagenesis are listed in Table 3.

**Table 2:** Primers used

Name of primer	Sequence
DnaA-box1CA-TG	F5’-CGGGGAATTTTGCTTATCTGCATTTAACTGG R5’-CAGATAAGCAAAATTCCCCGTCGCTGAG
DnaA-box1TT-GG	F5’-GACGGGGAATTTTGCGGATCTGCATTTAAC R5’-CAGATCCGCAAAATTCCCCGTCGCTGAG
DnaA-box3TA-GG	F5’- CGATATCTGAATTGTTAGGCATGTG R5’- CCTAACAATTCAGATATCGTTACCAGG
ORF2M-V	F5’- CCGGGGGATTGTGCTGAACGTCTTC R5’- CAATCCCCCGGAACAGCGTTTCCTC
DnaA-box8TT-GG	F5’- GTTGCAAGAAAACGTCGGACACAC R5’- CCGACGTTTTCTTGCAACAGCGGC
ORF111L-W	F5’-GCTTCACCTCCCGTTTGGATTTC R5’-CAAACGGGAGGTGAAGCCCACC
*bla*	R5’- AGCCATACCAAACGACGAGC
	R5’- TCAGCAATAAACCAGCCAGC
*terC*	R5’-TCCTCGCTGTTTGTCATCTT
	R5’-GGTCTTGCTCGAATCCCTT
*dnaA*	F5’-GCTCTAGAGGTGTCACTTTCGC
	R5’-GGGGTACCCGCGATGACAATGTTCTG
*repA*	F5’-AACTGCAGGGTGACTGATCTTCAACAAAC
	R5’-CGGGATCCTCGGGGGAAGCTGTGGCCAGC

**Table 3:** Mutations in DnaA-boxes and ORFs of the MiniR1–1 plasmid

Plasmid	Location of mutation	Sequence	
		Before mutagenesis	After mutagenesis
pbox1CA-TG	DnaA-box1	TTATCCACA	TTATCtgCA (DnaA-box1a)
pbox1TT-GG	DnaA-box1a	TTATCtgCA	ggATCtgCA
pbox3TA-GG	DnaA-box3	TTATACATG	TTAggCATG
pORF2M-V	DnaA-box4	TTATGCTGA	TTgTGCTGA
pbox8TT-GG	DnaA-box8	TCTTACACA	TCggACACA
pORF111L-W	ORF1	TTGATT	TgGATT

Mutations in DnaA-boxes and ORFs of the MiniR1–1 plasmid were introduced using primers listed in Table 2 and the ‘Easy mutagenesis system’ from Transgen biotech. Positions of the mutations and sequences before and after mutagenesis are listed. Lowercase letters indicate changed nucleotides

### Copy number determination of the plasmids by Q-PCR

Exponentially growing cells in ABT medium were collected, and then heated at 95 °C in 50 μL ddH_2_O for 10 min, and diluted serially in ddH_2_O prior to Q-PCR [26].

The ampicillin resistance gene (*bla*) in plasmids was used to determine the copy number of plasmid relative to the dosage of chromosomal *terC* site. The *bla* and *terC* specific primers for Q-PCR were designed using PrimerQuest online tool, and listed in Table 2.

The Q-PCR assay was performed in a LightCycter 480 II Real-Time PCR System (Roche, Switzerland) using SYBR®*Premix ExTaq*™II kit (TliRNaseH Plus) (TaKaRa, Japan). After an initial denature at 95 °C for 30 s, 40 cycles of 95 °C for 10 s, 60 °C for 20 s were used in the assay. Then melting curves were performed immediately as described previously [27]. The value of Ct from each reaction could automatically be given. Using the concentrations of template (the X axis) and threshold cycles (the Y axis), the relative standard curve was obtained. The amplification efficiency (E) was then calculated by the eq. 1, and the gene dosage was determined using the eq. 2 as described previously [26]. The copy number of plasmid relative to the *terC* site in each strain was then calculated.

1 $$ \mathrm{E}={10}^{\left(\hbox{-} 1/\mathrm{slope}\right)} $$

2 $$ \mathrm{PCN}={\left(\mathrm{Ec}\right)}^{\mathrm{Ctc}}/{\left(\mathrm{Ep}\right)}^{\mathrm{Ctp}} $$

### Bacterial two hybrid analysis

Plasmids and strain used in the bacterial two hybrid system (BCATH) are listed in Table 1. When two proteins interact, the T18 and T25 fragments can be combined together to catalyze the formation of cAMP. The synthesized cAMP activates the expression of the *lacZ* reporter gene, forming the blue colonies on plates containing X-gal and IPTG, whereas two proteins that do not interact will form white colonies. The BTH101 cells with a pair of plasmids expressing the proteins tested for interaction were cultured as previously described [27].

## Results

### The presence of MiniR1–1 leads to an accumulation of the D-period cells

**Fig. 1:**
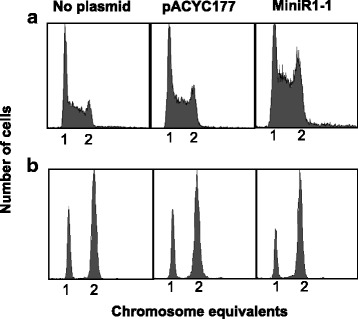
The presence of MiniR1–1 plasmid leads to an accumulation of the D-period cells. **a** Wild-type MG1655 cells without or with pACYC177 or MiniR1–1 plasmids were grown in ABT medium at 30 °C and fixed in 70% ethanol in the log phase. The cell cycle parameters were measured by flow cytometry. **b** Exponentially growing cells described in (**a**) were treated with rifampicin and cephlexin for 3–5 generations and analysed by flow cytometry. A total of 10,000 cells were included in each measurement

Analogous to the eukaryotic G1, S and M phase, slowly growing *Escherichia coli* cells can be classified into three stages of the cell cycle, i.e. B-, C- and the D-period, respectively [19, 28]. The B-period is the time between cell birth and initiation of chromosome replication; the C-period indicates the time required for chromosome replication; and the D-period is the time taken for cell division after chromosome segregation [28]. To see if the presence of MiniR1–1 plasmid affects the *E. coli* cell cycle, wild-type MG1655 cells were transformed with plasmids MiniR1–1 or pACYC177, respectively. The resultant transformants and the wild-type cells were exponentially grown at 30 °C in ABT medium as described in Methods [20]. The cell cycle parameters were measured by flow cytometry. In the wild-type MG1655 cell culture, 39% of the cells were in B-, 48% in C-, and 13% in the D-period with a doubling time of 196 min, whereas the presence of MiniR1–1 plasmid changed the distribution of cells to 28% in B-, 41% in C-, and 31% in the D-period with a doubling time of 195 min (Fig. 1a, Tables 4 and 5). The cell cycle parameters were not found to change due to different concentrations of ampicillin used in the medium (Additional file 2: Figure S2, Additional file 3 : Table S1). It is clear that the presence of MiniR1–1 plasmid increased the proportion of D-period cells, and that the proportions of cells in the B- and C-period were less than in the wild-type cells. The doubling time remained the same as that of the wild-type cells (Table 5). The presence of control plasmid pACYC177 resulted in a cell cycle distribution close to that of the wild-type with slightly more C- and D- period cells, but less B-period cells (Fig. 1a, Table 4). These results indicate that the presence of MiniR1–1 plasmid leads to an accumulation of the D-period cells.

**Table 4:** The presence of MiniR1–1 leads to accumulation of the D-period cells in slow growth

Strain	Cell cycle distribution (%)		
	B-period	C-period	D-period
MG1655	39(±1.5)	48(±1.5)	13(±1.0)
MG1655/pACYC177	32(±1.0)	54(±1.5)	14(±1.5)
MG1655/MiniR1–1	28(±1.0)	41(±1.0)	31(±1.0)
MG1655/pbox1CA-TG	27(±1.5)	51(±1.5)	22(±1.5)
MG1655/pbox1TT-GG	30(±1.0)	47(±1.5)	23(±1.0)
MG1655/pbox3TA-GG	25(±2.0)	49(±1.5)	26(±1.5)
MG1655/pORF2M-V	No growth		
MG1655/pbox8TT-GG	25(±1.1)	51(±1.1)	24(±1.5)
MG1655/pORF111L-W	21(±1.5)	51(±1.0)	28(±1.5)

Wild-type MG1655 cells carrying the plasmids listed above were grown at 30 °C in ABT medium, harvested at OD_450_ = 0.15, and analysed by flow cytometry as described in Methods. The proportion of cells in different periods of the cell cycle were calculated as described in Methods. The values are the average of three experiments, the standard errors are as shown in brackets

**Table 5:** The presence of MiniR1–1 results in an increase in cell size

Strain	Cell size (μm)	Doubling time (min)
MG1655	2.2(±0.1)	196(±1.5)
MG1655/pACYC177	2.2(±0.2)	185(±1.5)
MG1655/MiniR1–1	2.7(±0.1)	195(±1.5)
MG1655/pbox1CA-TG	3.2(±0.4)	200(±1.1)
MG1655/pbox1TT-GG	3.3(±0.2)	229(±1.0)
MG1655/pbox3TA-GG	2.5(±0.3)	198(±1.5)
MG1655/pORF2M-V	No growth	
MG1655/pbox8TT-GG	2.0(±0.1)	198(±1.1)
MG1655/pORF111L-W	2.8(±0.1)	188(±1.5)

Wild-type MG1655 cells carrying the plasmids listed above were grown at 30 °C in ABT medium, harvested at OD_450_ = 0.15, and analyzed by fluorescence microscopy as described in Methods. The values are the average of three experiments, the standard errors are as shown in brackets

### MiniR1–1 delays cell division

As described in the previous paragraph, the presence of MiniR1–1 leads to an accumulation of the D-period cells, and this can be due to a delay in cell division. To test this possibility, the cell size of exponentially growing cells grown under the same conditions as described above were measured by microscopy. Compared with the size of wild-type MG1655 cells (2.2 μm), the presence of MiniR1–1 plasmid, but not of pACYC177 plasmid, led to an increase in cell size (2.7 μm) without changing the doubling time (Table 5), suggesting that the presence of MiniR1 delays cell division. It is shown that cell division depends on chromosome replication, but that chromosome replication occurs without cell division [29]. Thus, an inhibition of chromosome replication stops cell division and this prevents premature cell division [30]. It is reasonable to assume that the delay in cell division could be due to a delay in initiation of chromosome replication in the presence of MiniR1–1. To test this hypothesis, exponentially growing cells in the medium mentioned above were treated with rifampicin and cephalexin for 3–5 generations and then analyzed by flow cytometry. As shown in Fig. 1b, the cells with MiniR1–1 plasmid had less one-chromosome and more two-chromosome cells compared to cells without plasmid or cells with pACYC177 plasmid. The initiation pattern of chromosome replication was not dependent on concentration of ampicillin used in the medium (Additional file 2: Figure S2, Additional file 4: Table S2). The average number of chromosome per cell was increased to 1.7 in the presence of MiniR1–1 relative to 1.5 in the wild-type cell or presence of pACYC177 (Table 6), showing that the number of chromosomes per cells is slightly increased in the presence of MiniR1–1, suggesting that the presence of MiniR1–1 leads to early initiation of chromosome replication. We conclude that the delayed cell division in the presence of MiniR1–1 is not due to problems in initiation of chromosome replication.

**Table 6:** The presence of MiniR1–1 increases the average number of chromosome per cell

Strain	The cell distribution of 1 or 2 chromosome equivalents (%)		Average number of chromosome per cell
	One chromosome cells	Two chromosome cells	A. C.
MG1655	51(±1.0)	49(±1.0)	1.5
MG1655/pACYC177	52(±1.7)	48(±1.7)	1.5
MG1655/MiniR1–1	31(±2.1)	69(±2.1)	1.7
MG1655/pbox1CA-TG	29(±1.5)	71(±1.5)	1.7
MG1655/pbox1TT-GG	30(±1.5)	70(±1.5)	1.7
MG1655/pbox3TA-GG	31(±1.5)	69(±1.5)	1.7
MG1655/pORF2M-V	No growth		
MG1655/pbox8TT-GG	30(±1.0)	70(±1.0)	1.7
MG1655/pORF111L-W	28(±2.1)	72(±2.1)	1.7

Wild-type MG1655 cells carrying the plasmids listed above were grown at 30 °Cin ABT medium, harvested at OD_450_ = 0.15, and analysed by flow cytometry as described in Methods. The proportion of cells in different periods of the cell cycle were calculated as described previously [20]. The values are the average of three experiments, the standard errors are as shown in brackets

### Complete nucleotide sequence of the MiniR1–1 plasmid

**Fig. 2:**
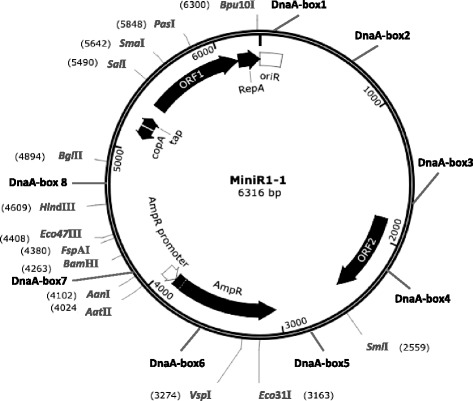
Genetic map and restriction sites of the MiniR1–1 plasmid. Using primers targeting the *datA* sequence in pMOR1 and pMOR1 as template, the complete genome of the MiniR1–1 plasmid was sequenced as described in Methods. The MiniR1–1 plasmid is a circular double stranded DNA molecule, consisting of 6316 bp. DnaA-boxes, *oriR*1, ORFs and genes are as indicated. The arrows represent orientation of transcription. Restriction sites of the MiniR1–1 plasmid were determined by BVTech Plasmid software which is available on the internet (http://www.biovisualtech.com/bvplasmid/downloadpl.htm). The restriction enzymes and sites are as illustrated

The *oriR*1 region of R1 plasmid contains a DnaA-box [10]. Thus, titration of DnaA proteins to DnaA-boxes in MiniR1–1 could be the reason why the presence of MiniR1–1 delays cell division, since excess *datA* sites delay both initiation of chromosome replication and cell division [31]. The *datA* site is a 1 kb region carrying five DnaA-boxes, binding a large amount of initiator DnaA protein [32]. To check if the MiniR1–1 plasmid contains additional potential DnaA-boxes besides one in *oriR*1, we sequenced the MiniR1–1 plasmid genome using primers targeting the *datA* sequence in pMOR1 as described in Methods. The MiniR1–1 plasmid is a circular double stranded DNA molecule, consisting of 6316 bp (Fig. 2, Additional file 5: Figure S3). The plasmid has a 188 bp *oriR*1 region [33] situated close to the *repA*, *tap* and *copA* genes. The *tap* gene encodes a small leader peptide [8] and the *copA* gene is transcribed in the opposite direction compared to the *tap* transcriptions [3]. The *repA* and *tap* genes are separated by ORF1 and there is an ORF2 between *oriR*1 and the *bla* gene. ORF1 is predicted to encode a protein which is a hypothetical protein (SEEMU129_23570) of *Salmonella enterica subsp. enteric aserovar Muenchen* str. RKS4129 and a hypothetical protein (pU302L_074) of *Salmonella enterica subsp. enterica serovar Typhimurium*. ORF2 is predicted to encode fimbrial-like adhesin protein of *E. coli*. Eight DnaA-boxes found are situated as indicated in the MiniR1–1 genetic map (Fig. 2). One consensus DnaA-box is in *oriR*1, one DnaA-box with one mismatch and six DnaA-boxes with two mismatches are also shown in the genetic map, namely, DnaA-box1, box2, box3, box4, box5, box6, box7 and box8, respectively (Fig. 2).

We also analyzed restriction sites of MiniR1–1 using software BVTech Plasmid which is available on the internet (http://www.biovisualtech.com/bvplasmid/downloadpl.htm). The MiniR1–1 plasmid has 36 restriction sites in total. There are 4 restriction sites in the downstream region of ORF2, 3 in *bla* gene, 13 between *copA* and *bla* genes, and 16 in ORF1 (Fig. 2).

### Mutations in DnaA-boxes increases the copy number of MiniR1–1

To interfere with titration (binding) of DnaA to DnaA-boxes, mutations were introduced into the MiniR1–1 DnaA-boxes by using site directed mutagenesis. A pair of primers (DnaA-box1CA-TG) was used to change two nucleotides in DnaA-box1, constructing plasmid pbox1CA-TG. Further, using pbox1CA-TG as template and primers DnaA-box1TT-GG, two more nucleotide changes were introduced into DnaA-box1, resulting in a plasmid with scrambled DnaA-box1, pbox1TT-GG. Also a pair of primers (DnaA-box3TA-GG) was used to introduce mutations in DnaA-box3. The ORF1–11 L-W or ORF2M-V primer pairs were designed to change one amino acid in ORF1 or ORF2. Two nucleotides in DnaA-box8 were changed by use of the DnaA-box8TT-GG primers. The resultant plasmids and nucleotide changes after mutagenesis are listed in Table 3 and the primers used are listed in Table 2.

To determine the relative copy number of MiniR1–1, slowly growing MG1655 cells harboring a single plasmid (Table 3) in ABT medium were collected and subsequently used as template for Q-PCR analysis [26] as described in Methods. We found that the relative copy number of the control plasmid pACYC177 per the chromosomal *terC* site was 11 whereas that of MiniR1–1 was 1 (Table 7). The relative copy number of MiniR1–1 was increased to 6 copies per *terC* upon mutations in the MiniR1–1 DnaA-box1, it was 5 copies responding to mutations in DnaA-boxes8 and 2 copies to mutations in DnaA-box3 (Table 7). It should be noted that mutations in ORF1 did not change the plasmid copy number. The results indicate that mutations in the MiniR1–1 DnaA-boxes increase the copy number of MiniR1–1, suggesting that binding of DnaA to DnaA-boxes in MiniR1–1 negatively controls initiation of the plasmid replication.

**Table 7:** Mutations in DnaA-boxes affect copy number of the MiniR1–1 plasmid

Strain	Relative copy number of plasmid
MG1655/pACYC177	11 (±3.0)
MG1655/MiniR1–1	1 (±2.7)
MG1655/pbox1CA-TG	6 (±1.3)
MG1655/pbox1TT-GG	6 (±1.7)
MG1655/pbox3TA-GG	2 (±0.7)
MG1655/pORF2M-V	Not determined
MG1655/pbox8TT-GG	5 (±1.8)
MG1655/pORF111L-W	1 (±0.6)

Slowly growing wild-type MG1655 cells carrying individaul plasmid listed were collected for Q-PCR. Copy number of the plamids relative to the chromosomal *terC* site was determined by Q-PCR as described in Methods. The values are the average of three experiments, the standard errors are as shown in brackets

### The presence of MiniR1–1 with scrambled DnaA-box1 or mutated box8 affects cell size and growth rate

**Fig. 3:**
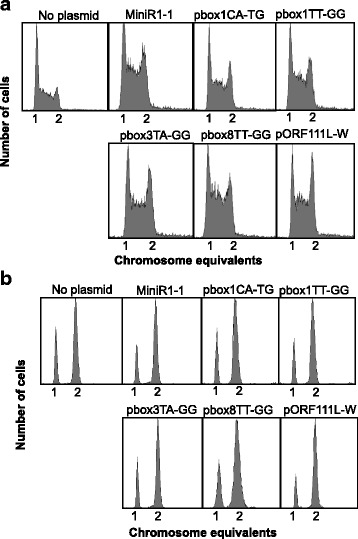
Mutations in the MiniR1–1 DnaA-boxes lead to a slight decrease in the number of D-period. The wild-type MG1655 cells containing plasmids as indicated were exponentially grown (**a**) or treated with rifampicin and cephalexin (**b**) in ABT medium at 30 °C. Cell cycle parameters were measured as described in the legend to Fig. 1. A total of 10,000 cells was included in each measurement

The subsequent cell cycle parameter measurement by flow cytometry showed that the cells harbouring plasmids with mutations in DnaA-box1, box3, box8 or ORF1 showed a slight increase in the proportion of C-period cells and a minor decrease in the proportion of D-period cells relative to the cells containing MiniR1–1 (Fig. 3a, Table 4). The cells maintained the initiation pattern of chromosome replication in the cells harbouring wild-type MiniR1–1 (Fig. 3b, Table 6). The presence of plasmid pbox1TT-GG (MiniR1–1 with scrambled DnaA-box1), particularly, did not affect initiation of chromosomal replication (Fig. 3b), indicating that scrambled DnaA-box1 in *oriR*1 does not affect chromosome replication.

Interestingly, the cells carrying MiniR1–1 with scrambled DnaA-box1 had a larger cell size (3.3 μm) and slower growth rate relative to cells with MiniR1–1 (Table 5). On the contrary, mutations in DnaA-box8 (pbox8TT-GG) led to a significant decrease in cell size (2.0 μm) relative to that in the presence of MiniR1–1 (2.7 μm) without affecting the growth rate (Table 5). The results suggest that the delay in cell division in the presence of MiniR1–1 may be, at least, partially due to titration of DnaA to DnaA-box1 and box8, and that DnaA-box1 functions differently from DnaA-box8.

### Mutations in ORF2 of MiniR1–1 affect cell survival

Unexpectedly, mutations in DnaA-box4, which is situated in ORF2 of MiniR1–1, led to cell death in AB minimal medium (Table 5), but the mutations allowed cell growth in rich media (LB) and ABTGcasa (see Methods). The mutations led to a change of methionine to valine in ORF2 which is a predicted *E. coli* fimbrial-like adhesin protein. The bacterial fimbrial–like adhesion protein is a macromolecular structure, mediating the attachment of bacteria to a surface [34]. The results indicate that mutations in ORF 2 of MiniR1–1 affect cell survival.

### The DnaA protein weakly interacts with the initiator RepA protein

**Fig. 4:**
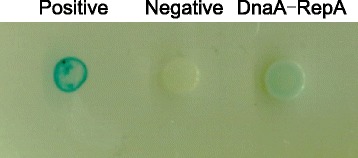
The DnaA protein weakly interacts with RepA protein in vivo. BTH101 cells were co-transformed with plasmid pairs encoding T18 and T25 respectively as negative control, then plated on the LB plates mentioned above with required antibiotics, incubated at 30 °C for 30 h. BTH101 cells were co-transformed with plasmid pairs encoding T18-TorR and T25-MreB respectively as positive control, proved previously. To exclude “false” blue colonies, 3 μl of the bacterial culture from each transformant after grown in LB for 2 h was mounted the same plate as described above, incubated at 30 °C for 30 h. All transformants illustrating different protein-protein interactions were simultaneously tested on one plate to have the same reaction condition. The blue bacterial halos indicate protein-protein interactions while the white halos show no interaction. Protein pairs under detection are as indicated

We show that mutations in DnaA-box1 in the *oriR* region significantly increase the copy number of MiniR1–1 plasmid, suggesting that binding of DnaA to DnaA-box1 decrease the initiation frequency of MiniR1–1 replication. It has been suggested that DnaA might interact with a RepA-*oriR* pre-initiation complex in the absence of DnaA-box in *oriR* to trigger initiation of replication [10, 12]. It could be possible that DnaA might interact with RepA at *oriR* to strictly control initiation of the MiniR1–1 replication. To check this possibility, we analyzed the interaction of DnaA with RepA in vivo by bacterial two hybrid system [35]. When two proteins physically interact in the bacterial two-hybrid system, the reporter *lacZ* gene can be expressed in a cAMP/CRP (cAMP receptor protein)-dependent way [35]. This expression can produce blue colonies, on LB plates containing X-gal, whereas white colonies will appear upon no interaction between the proteins. The cells expressing DnaA and RepA resulted in very weak blue colonies whereas the positive control (TorR-MreB interaction) gave rise to blue colonies [27], and the negative control cell colonies remained white (Fig. 4). The results indicate that DnaA interacts with RepA weakly in vivo (Fig. 4), suggesting that direct interaction between DnaA and RepA may be required for fine control of the MiniR1–1 replication.

## Discussion

### DnaA is a negative factor for replication of the MiniR1–1 plasmid

RepA-driven initiation of the R1 plasmid replication is more efficient in the presence of DnaA [10, 12]. However, RepA initiates replication of the R1 plasmid at *oriR* in vivo in the absence of DnaA [13, 14]. As shown in Fig. 2, DnaA-box1 is in *oriR*1, and mutations in DnaA-box1 increases copy number of the plasmid from 1 to 6 copies per *terC* (Table 7). The result suggests that the binding of DnaA to DnaA-box1 negatively controls initiation of the MiniR1–1 replication as a possible result of DnaA interfering with the function of RepA. DnaA-box8 is immediately downstream of the *copA* gene which transcribes antisense RNA, and the RNA inhibits RepA-translation. Mutations in DnaA-box8 leads also to a significant increase in copy number of the MiniR1–1 plasmid (Table 7). Interestingly, mutations in DnaA-box3, which is situated upstream of ORF2, slightly increase copy number of the plasmid (only 2 copies per cell). Clearly, the effect of DnaA-box3 is different from that of the DnaA-box1 and DnaA-box8 on initiation of the plasmid replication. It is most likely that binding of DnaA to DnaA-box8 may limit CopA-RNA synthesis due to a close localization of DnaA-box8 to the *copA* gene, and consequently promote RepA-translation. The enhanced RepA production, in turn, initiates replication of MiniR1–1 more efficiently. Therefore, we conclude that DnaA is a negative factor for control of the MiniR1–1 replication, and may balance copy number of the plasmid through interacting with RepA (Fig. 4).

### Titration of DnaA to the MiniR1–1 DnaA-boxes affect cell size and growth rate

The cell size of *E.coli* is highly correlated with the growth rate, i.e. cells are larger when growing under fast growth conditions compared to poor growth conditions. Indeed, it was shown that the *E. coli* wild-type cells were larger with fast growth rate in rich medium (LB) and smaller with slower growth rate in ABTGcasa medium (not as rich as LB). Further, wild-type cells were found to be even smaller with very slow growth rate in poor medium (ABT) [20]. Cells harbouring MiniR1–1 was found to be larger relative to the cells without MiniR1–1, but the growth rates of the two strains were the same. The result suggests that the cells with MiniR1–1 have difficulties with cell division since the growth rate is the same as that of the cells without MiniR1–1. It has been shown that growth rate is not always a measure for initiation of replication and cell division [20]. We understand that cell division might be delayed when a strain has a larger cell size with the same or slower growth rate relative to those of the control strain. The conclusion is supported by the fact that the strain with MiniR1–1 had more D-period cells compared with the strain without the plasmid. Obviously, the accumulation of the D-period cells indicates a delay of cell division when the growth rate is the same.

As discussed above, the presence of MiniR1–1 plasmid delays cell division without delaying initiation of chromosome replication (Fig. 1). How does the presence of MiniR1–1 delay cell division? Mutations in the MiniR1–1 DnaA-box1 result in further delay in cell division (Table 5) and an increase in copy number of the plasmid (Table 7). The delayed cell division could be explained by the increased dosage of the MiniR1–1 DnaA-boxes since high dosage of DnaA-boxes would lead to a large decrease in DnaA availability as a result of titration of more DnaA to DnaA-boxes. A decrease in DnaA availability delays cell division [31]. However, in contrast, mutations in DnaA-box8 produce smaller cells with increased copies of MiniR1–1 but not changing growth rate, suggesting that mutated DnaA-box8 facilitates cell division (Tables 5 and 7). Interestingly, mutations in DnaA-box3 do not change the delay of cell division due to the presence of MiniR1–1. It is likely that low copy MiniR1–1 may delay the *E. coli* cell division due to problems in partition of plasmid copies.

## Conclusions

The presence of MiniR1–1 leads to a delay of the *E. coli* cell division in the slow growth condition. Mutations in the MiniR1–1 DnaA-boxes not only affect cell size and growth rate but also increase the copy number of the plasmid. It is likely that titration of DnaA to DnaA-boxes negatively controls replication of the MiniR1–1 plasmid through interacting with the initiator protein RepA.

## Additional files

Additional file 1: **Figure S1.** Illustration for determination of the B-, C and D-period in an exponentially growing cluture. (DOCX 215 kb)

Additional file 2: **Figure S2.** The concentrations of ampicillin do not affect chromosomal replication pattern. (DOCX 75 kb)

Additional file 3: **Table S1.** The concentrations of ampicillin do not affect the cell-cycle parameters. (DOCX 14 kb)

Additional file 4: **Table S2.** The concentrations of ampicillin do not affect the average number of origin per cell. (DOCX 15 kb)

Additional file 5: **Figure S3.** Sequencing results for the MiniR1–1 plasmid. (PDF 89 kb)

## Abbreviations

*aad*: streptomycin/spectinomycin
*bla*: ampicillin
Bp: base pair
cAMP: cyclic adenosine monophosphate
*cat*: chloramphenicol
CRP: catabolite activator protein
E: efficiency
*E. coli*: *Escherichia coli*
IPTG: Isopropyl β-D-1-thiogalactopyranoside
*neo*: kanamycin
OD: optical density
*oriR*: origin for replication
PCN: copy number of plasmid
Q-PCR: quantitative polymerase chain reaction
*sul*: sulfonamide
Tap: translational activator peptide
