# Supplementary material for: Mutations of DnaA-boxes in the oriR region increase replication frequency of the MiniR1–1 plasmid
Source: BMC Microbiol. 2018 Apr 3;18:27. doi: 10.1186/s12866-018-1162-3 (PMC5883639; doi:10.1186/s12866-018-1162-3)
Supplement: Supplementary file 1 — Figure S1. Illustration for determination of the B-, C and D-period in an exponentially growing cluture. (DOCX 215 kb) [file 12866_2018_1162_MOESM1_ESM.docx]

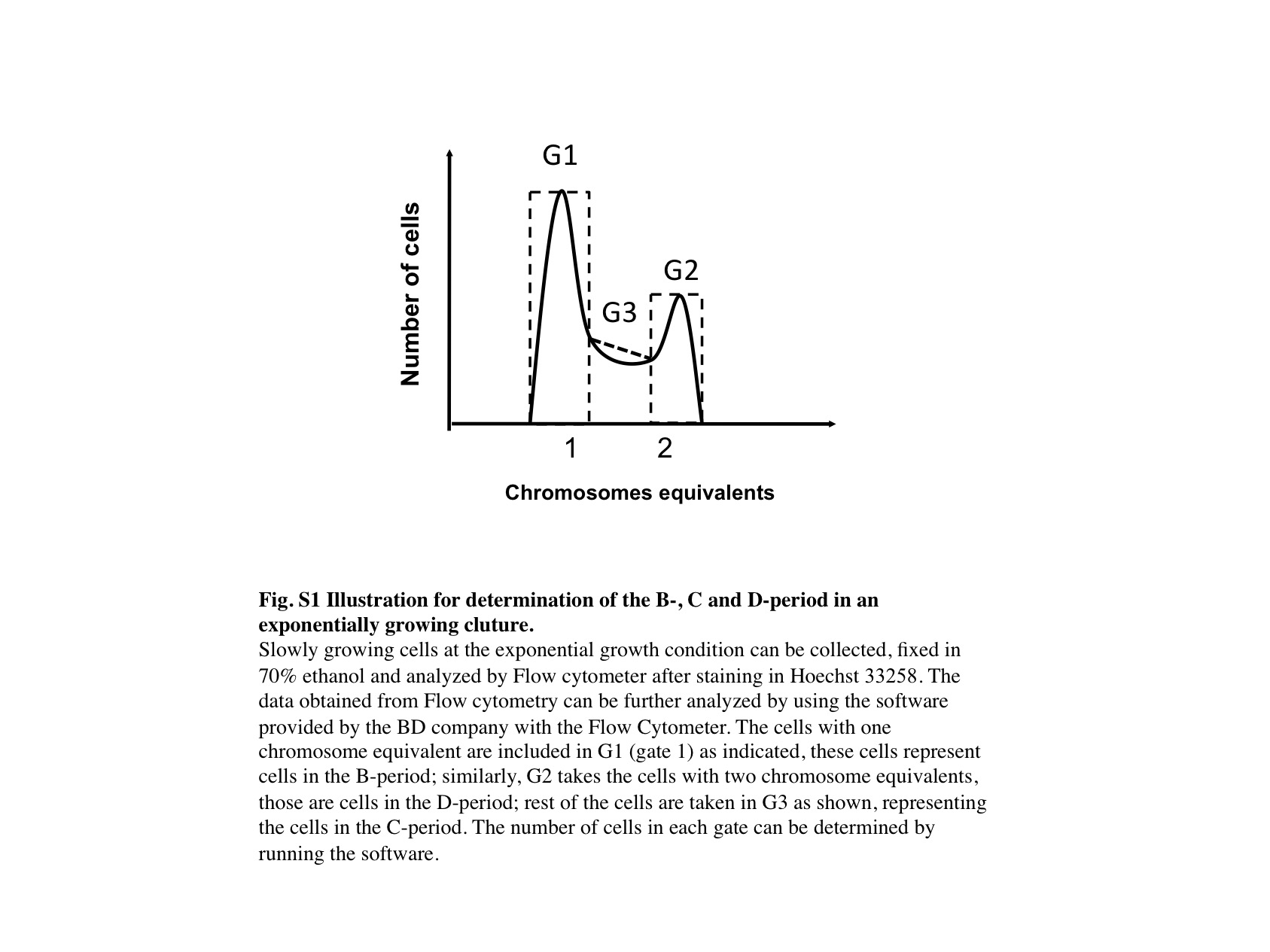


Figure S1. Illustration for determination of the B-, C and D-period in an exponentially growing cluture. Slowly growing cells at the exponential growth condition can be collected, fixed in 70% ethanol and analyzed by Flow cytometer after staining in Hoechst 33258. The data obtained from Flow cytometry can be further analyzed by using the software provided by the BD company with the Flow Cytometer. The cells with one chromosome equivalent are included in G1 (gate 1) as indicated, these cells represent cells in the B-period; similarly, G2 takes the cells with two chromosome equivalents, those are cells in the D-period; rest of the cells are taken in G3 as shown, representing the cells in the C-period. The number of cells in each gate can be determined by running the software.
