# Supplementary material for: Mutations of DnaA-boxes in the oriR region increase replication frequency of the MiniR1–1 plasmid
Source: BMC Microbiol. 2018 Apr 3;18:27. doi: 10.1186/s12866-018-1162-3 (PMC5883639; doi:10.1186/s12866-018-1162-3)
Supplement: Supplementary file 2 — Figure S2. The concentrations of ampicillin do not affect chromosomal replication pattern. (DOCX 75 kb) [file 12866_2018_1162_MOESM2_ESM.docx]

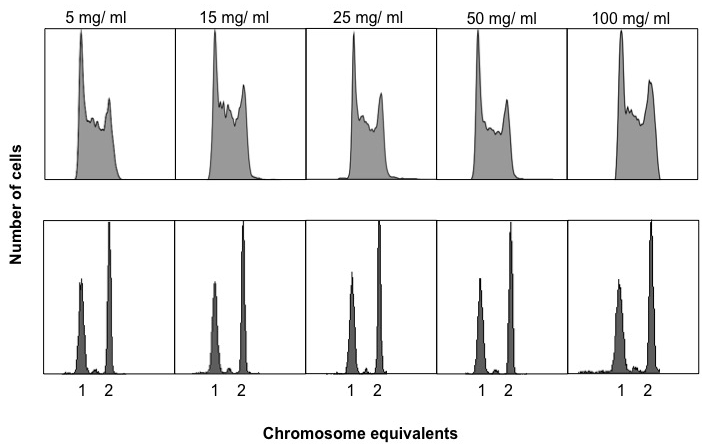


Figure S2. The concentrations of ampicillin do not affect chromosomal replication pattern. Exponentially growing cells (A) in ABT medium were sampled or treated with rifampicin and cephalexin (B) as described in the legend to Fig. 1.
