# Supplementary material for: Mutations of DnaA-boxes in the oriR region increase replication frequency of the MiniR1–1 plasmid
Source: BMC Microbiol. 2018 Apr 3;18:27. doi: 10.1186/s12866-018-1162-3 (PMC5883639; doi:10.1186/s12866-018-1162-3)
Supplement: Supplementary file 3 — Table S1. The concentrations of ampicillin do not affect the cell-cycle parameters. (DOCX 14 kb) [file 12866_2018_1162_MOESM3_ESM.docx]

Table S1. The concentrations of ampicillin do not affect the cell-cycle parameters

| Concentrations of ampicillin | Cell cycle distribution (%) | | |
| --- | --- | --- | --- |
|  | B-period | C-period | D-period |
| 5 mg/ml | 28(±2.0) | 44(±3.0) | 28(±1.0) |
| 15 mg/ml | 30(±3.0) | 49(±2.0) | 21(±3.0) |
| 25 mg/ml | 30(±2.0) | 44(±1.0) | 26(±2.0) |
| 50 mg/ml | 32(±1.0) | 44(±4.0) | 24(±1.0) |
| 100 mg/ml | 31(±2.0) | 45(±1.0) | 23(±2.0) |
