# Supplementary material for: Mutations of DnaA-boxes in the oriR region increase replication frequency of the MiniR1–1 plasmid
Source: BMC Microbiol. 2018 Apr 3;18:27. doi: 10.1186/s12866-018-1162-3 (PMC5883639; doi:10.1186/s12866-018-1162-3)
Supplement: Supplementary file 4 — Table S2. The concentrations of ampicillin do not affect the average number of origin per cell. (DOCX 15 kb) [file 12866_2018_1162_MOESM4_ESM.docx]

| Concentrations of ampicillin (treated with Rif and Cpx) | The cell distribution of 1 or 2 chromosome equivalents (%) | | Average number of chromosome per cell |
| --- | --- | --- | --- |
|  | 1 | 2 | A. C |
| 5 mg/ml | 39(±2.1) | 61(±2.7) | 1.61 |
| 15 mg/ml | 38(±2.5) | 62(±2.5) | 1.62 |
| 25 mg/ml | 36(±2.5) | 64(±2.5) | 1.64 |
| 50 mg/ml | 35(±2.5) | 65(±1.5) | 1.65 |
| 100 mg/ml | 36(±2.8) | 64(±1.1) | 1.64 |

Table S2. The concentrations of ampicillin do not affect the average number of origin per cell.
