# Supplementary material for: Mutations of DnaA-boxes in the oriR region increase replication frequency of the MiniR1–1 plasmid
Source: BMC Microbiol. 2018 Apr 3;18:27. doi: 10.1186/s12866-018-1162-3 (PMC5883639; doi:10.1186/s12866-018-1162-3)
Supplement: Supplementary file 5 — Figure S3. Sequencing results for the MiniR1–1 plasmid. (PDF 89 kb) [file 12866_2018_1162_MOESM5_ESM.pdf]

5′ TTGCTTATCCACATTTAACTGCAAGGGACTTCCCCATAAGGTTACAACCGTTCATGTCAT 60
3′ AACGAATAGGTGTAAATTGACGTTCCCTGAAGGGGTATTCCAATGTTGGCAAGTACAGTA

oriR

DnaA box 1

AAAGCGCCAGCCGCCAGTCTTACAGGGTGCAATGTATCTTTTAAACACCTGTTTATATCT 120
TTTCGCGGTCGGCGGTCAGAATGTCCCACGTTACATAGAAAATTTGTGGACAAATATAGA

oriR

CCTTTAAACTACTTAATTACATTCATTTAAAAAGAAAACCTATTCACTGCCTGTCCTGTG 180
GGAAATTTGATGAATTAATGTAAGTAAATTTTTCTTTTGGATAAGTGACGGACAGGACAC

oriR

GACAGACAGATATGCACCTCCCACCGCAAGCGGCGGGCCCCGACCGGAGCCACTTTAGTT 240
CTGTCTGTCTATACGTGGAGGGTGGCGTTCGCCGCCCGGGGCTGGCCTCGGTGAAATCAA

ACAACACACAAAAACAACCTCCAGAAAAACCCCGGTCCAGCGCAGAACCGAAACCACAAA 300
TGTTGTGTGTTTTTGTTGGAGGTCTTTTTGGGGCCAGGTCGCGTCTTGGCTTTGGTGTTT

GCCCCTCCCTCATAACTGAAAAGCGGCCCCGCCCCGGCCCAAAGGGCCGGAACAGAGTCG 360
CGGGGAGGGAGTATTGACTTTTCGCCGGGGCGGGGCCGGGTTTCCCGGCCTTGTCTCAGC

CTTTTAATTATGAATGTTGTAACTACATCTTCATCGCTGTCAGTCTTCTCGCTGGAAGTT 420
GAAAATTAATACTTACAACATTGATGTAGAAGTAGCGACAGTCAGAAGAGCGACCTTCAA

CTCAGTACACGCTCGTAAGCGGCCCTCACGGCCCGCTAACGCGGAGATACGCCCCGACTT 480
GAGTCATGTGCGAGCATTCGCCGGGAGTGCCGGGCGATTGCGCCTCTATGCGGGGCTGAA

CGGGTAAACCCTCGTCGGGACCACTCCGACCGCGCACAGAAGCTCTCTCATGGCTGAAAG 540
GCCCATTTGGGAGCAGCCCTGGTGAGGCTGGCGCGTGTCTTCGAGAGAGTACCGACTTTC

CGGGTATGGTCTGGCAGGGCTGGGGATGGGTAAGGTGAAATCTATCAATCAGTACCGGCT 600
GCCCATACCAGACCGTCCCGACCCCTACCCATTCCACTTTAGATAGTTAGTCATGGCCGA

TACGCCGGGCTTCGGCGGTTTTACTCCTGTATCATATGAAACAACAGAGTGCCGCCTTCC 660
ATGCGGCCCGAAGCCGCCAAAATGAGGACATAGTATACTTTGTTGTCTCACGGCGGAAGG

ATGCCGCTGATGCGGCATATCCTGGTAACGATATCTGAATTGTTATACATGTGTATATAC 720
TACGGCGACTACGCCGTATAGGACCATTGCTATAGACTTAACAATATGTACACATATATG

DnaA box2

```
GTGGTAATGACAAAAATAGGACAAGTTAAAAATTTACAGGCGATGCAATGATTCAAACAC
                                                              780
CACCATTACTGTTTTTATCCTGTTCAATTTTTAAATGTCCGCTACGTTACTAAGTTTGTG

GTAATCAATATCTGCAGTTTATGCTGGTTATGCTGGCTGCATGGGGCATTAGTTGGGGAG
                                                              840
CATTAGTTATAGACGTCAAATACGACCAATACGACCGACGTACCCCGTAATCAACCCCTC

CCAGATTTGTCATGGAGCAGGCCGTTCTGCTTTATGGATCAGGAAAAAACTATTTGTTCT
                                                              900
GGTCTAAACAGTACCTCGTCCGGCAAGACGAAATACCTAGTCCTTTTTTGATAAACAAGA

TCAGTCATGGTACTGTTCTGATGTACCTGCTGTGTGTTTTCCTGGTATACCGCCGTTGGA
                                                              960
AGTCAGTACCATGACAAGACTACATGGACGACACACAAAAGGACCATATGGCGGCAACCT

TAGCTCCGCTACCGGTCGTTGGTCAGCTGCGCAACGTTGGCGTACCGTGGCTGGTCGGTG
                                                              1020
ATCGAGGCGATGGCCAGCAACCAGTCGACGCGTTGCAACCGCATGGCACCGACCAGCCAC

CGATGGCCGTGGTGTATGTCGGTGTATTTCTGCTCGGTAAGGCGCTGGCTCTGCTGCTGA
                                                              1080
GCTACCGGCACCACATACAGCCACATAAAGACGAGCCATTCCGCGACCGAGACGACGACT

GCCATTTATGACGAAACTTTTTGCCGATAAGTCCATACCTGACGTGATCCTGACGTTGCT
                                                              1140
CGGTAAATACTGCTTTGAAAAACGGCTATTCAGGTATGGACTGCACTAGGACTGCAACGA

GACCATCTTTATCCTTGCCCCGTTGAATGAGGAAACGCTGTTCCGGGGGATTATGCTGAA
                                                              1200
CTGGTAGAAATAGGAACGGGGCAACTTACTCCTTTGCGACAAGGCCCCCTAATACGACTT

CGTCTTCCGTTCACGTACTGCTGGACGATGTGGCTGGGGGCGCTGATAACGTCGTTGTTG
                                                              1260
GCAGAAGGCAAGTGCATGACGACCTGCTACACCGACCCCCGCGACTATTGCAGCAACAAC

TTCGTCGCCGCGCACAGCCAGTATCAGAACCTGCTGACACTGGCAGAACTGTTCCTGGTG
                                                              1320
AAGCAGCGGCGCGTGTCGGTCATAGTCTTGGACGACTGTGACCGTCTTGACAAGGACCAC

GGGTTGATTACATCAGTGGCCAGGATCAGAAGTGGTGGCCTGCTGCTGCCAAAGCCCTCC
                                                              1380
CCCAACTAATGTAGTCACCGGTCCTAGTCTTCACCACCGGACGACGACGGTTTCGGGAGG

CTCATAACTGAAAAGCGGCCCGCCCCGGCCCAAAGGGCCGGAACAGAGTCGCTTTTAAAT
                                                              1440
GAGTATTGACTTTTCGCCGGGCGGGGCCGGGTTTCCCGGCCTTGTCTCAGCGAAAATTTA

TATGAATGTTGTAACTACATCTTCATCGCTGTCAGTCTTCTCGCTGGAAGTTCTCAGTAC
                                                              1500
ATACTTACAACATTGATGTAGAAGTAGCGACAGTCAGAAGAGCGACCTTCAAGAGTCATG

ACGCTCGTAAGCGGCCCTCACGGCCCGCTAACGCGGAGATACGCCCCGACTTCGGGTAAA
                                                              1560
TGCGAGCATTCGCCGGGAGTGCCGGGCGATTGCGCCTCTATGCGGGGCTGAAGCCCATTT
```

```
CCCTCGTCGGGACCACTCCGACCGCGCACAGAAGCTCTCTCATGGCTGAAAGCGGGTATG    1620
GGGAGCAGCCCTGGTGAGGCTGGCGCGTGTCTTCGAGAGAGTACCGACTTTCGCCCATAC

GTCTGGCAGGGCTGGGGATGGGTAAGGTGAAATCTATCAATCAGTACCGGCTTACGCCGG    1680
CAGACCGTCCCGACCCCTACCCATTCCACTTTAGATAGTTAGTCATGGCCGAATGCGGCC

GCTTCGGCGGTTTTACTCCTGTATCATATGAAACAACAGAGTGCCGCCTTCCATGCCGCT    1740
CGAAGCCGCCAAAATGAGGACATAGTATACTTTGTTGTCTCACGGCGGAAGGTACGGCGA

GATGCGGCATATCCTGGTAACGATATCTGAATTGTTATACATGTGTATATACGTGGTAAT    1800
CTACGCCGTATAGGACCATTGCTATAGACTTAACAATATGTACACATATATGCACCATTA
                                            DnaA box3

GACAAAAATAGGACAAGTTAAAAATTTACAGGCGATGCAATGATTCAAACACGTAATCAA    1860
CTGTTTTTATCCTGTTCAATTTTTAAATGTCCGCTACGTTACTAAGTTTGTGCATTAGTT
                                                  ORF2

TATCTGCAGTTTATGCTGGTTATGCTGGCTGCATGGGGCATTAGTTGGGGAGCCAGATTT    1920
ATAGACGTCAAATACGACCAATACGACCGACGTACCCCGTAATCAACCCCTCGGTCTAAA
                              ORF2

GTCATGGAGCAGGCCGTTCTGCTTTATGGATCAGGAAAAAACTATTTGTTCTTCAGTCAT    1980
CAGTACCTCGTCCGGCAAGACGAAATACCTAGTCCTTTTTTGATAAACAAGAAGTCAGTA
                              ORF2

GGTACTGTTCTGATGTACCTGCTGTGTGTTTTCCTGGTATACCGCCGTTGGATAGCTCCG    2040
CCATGACAAGACTACATGGACGACACACAAAAGGACCATATGGCGGCAACCTATCGAGGC
                              ORF2

CTACCGGTCGTTGGTCAGCTGCGCAACGTTGGCGTACCGTGGCTGGTCGGTGCGATGGCC    2100
GATGGCCAGCAACCAGTCGACGCGTTGCAACCGCATGGCACCGACCAGCCACGCTACCGG
                              ORF2

GTGGTGTATGTCGGTGTATTTCTGCTCGGTAAGGCGCTGGCTCTGCCTGCTGAGCCATTT    2160
CACCACATACAGCCACATAAAGACGAGCCATTCCGCGACCGAGACGGACGACTCGGTAAA
                              ORF2

ATGACGAAACTTTTTGCCGATAAGTCCATACCTGACGTGATCCTGACGTTGCTGACCATC    2220
TACTGCTTTGAAAAACGGCTATTCAGGTATGGACTGCACTAGGACTGCAACGACTGGTAG
                              ORF2
```

```
TTTATCCTTGCCCCGTTGAATGAGGAAACGCTGTTCCGGGGGATTATGCTGAACGTCTTC   2280
AAATAGGAACGGGGCAACTTACTCCTTTGCGACAAGGCCCCCTAATACGACTTGCAGAAG
ORF2
DnaA box4

CGTTCACGGTACTGCTGGACGATGTGGCTGGGGGCGCTGATAACGTCGTTGTTGTTCGTC   2340
GCAAGTGCCATGACGACCTGCTACACCGACCCCCGCGACTATTGCAGCAACAACAAGCAG
ORF2

GCCGCGCACAGCCAGTATCAGAACCTGCTGACACTGGCAGAACTGTTCCTGGTGGGGTTG   2400
CGGCGCGTGTCGGTCATAGTCTTGGACGACTGTGACCGTCTTGACAAGGACCACCCCAAC
ORF2

ATTACATCAGTGGCCAGGATCAGAAGTGGTGGCCTGCTGCTGCCGGTATTGCTGCATATG   2460
TAATGTAGTCACCGGTCCTAGTCTTCACCACCGGACGACGACGGCCATAACGACGTATAC
ORF2

GAAGCAACCACGCTGGGTTTACTGTTTGGTTGAAAGTTATATTTTTATTAAACATTGTGC   2520
CTTCGTTGGTGCGACCCAAATGACAAACCAACTTTCAATATAAAAATAATTTGTAACACG
ORF2

                                                SmiI
GTTAAAGCCTGGTGTGTTTTTTTAGTGGATGTTATATTTAAATATAACTTTTATGGAGGT   2580
CAATTTCGGACCACACAAAAAAATCACCTACAATATAAATTTATATTGAAAATACCTCCA

GAAGAATGCATACCACCCGACTGAAGAGGGTTGGCGGCTCAGTTATGCTGACCGTCCCAC   2640
CTTCTTACGTATGGTGGGCTGACTTCTCCCAACCGCCGAGTCAATACGACTGGCAGGGTG
DnaA box5

CGGCACTGCTGAATGCGCTGTCTCTGGGCACAGATAATGAAGTTGGCATGGTCATTGATA   2700
GCCGTGACGACTTACGCGACAGAGACCCGTGTCTATTACTTCAACCGTACCAGTAACTAT

ATGGCCGGCTGATTGTTGAGCCGTACAGACGCCCGCAATATTCACTGGCTGAGCTACTGG   2760
TACCGGCCGACTAACAACTCGGCATGTCTGCGGGCGTTATAAGTGACCGACTCGATGACC

CACAGTGTGATCCGAATGCTGAAATATCAGCTGAAGAACGAGAATGGCTGGATGCACCGG   2820
GTGTCACACTAGGCTTACGACTTTATAGTCGACTTCTTGCTCTTACCGACCTACGTGGCC

CGACTGGTCAGGAGGAAATCTGACATGGAAAGAGGGGAAATCTGGCTTGTCTCGCTTGAT   2880
GCTGACCAGTCCTCCTTTAGACTGTACCTTTCTCCCCTTTAGACCGAACAGAGCGAACTA
```

```
CGGGGTCTGACGCTCAGTGGAACGAAAACTCACGTTAAGGGATTTTGGTCATGAGATTAT
                                                                2940
GCCCCAGACTGCGAGTCACCTTGCTTTTGAGTGCAATTCCCTAAAACCAGTACTCTAATA
```

```
CAAAAAGGATCTTCACCTAGATCCTTTTAAATTAAAAATGAAGTTTTAAATCAATCTAAA
                                                                3000
GTTTTTCCTAGAAGTGGATCTAGGAAAATTTAATTTTTACTTCAAAATTTAGTTAGATTT
```

```
GTATATATGAGTAAACTTGGTCTGACAGTTACCAATGCTTAATCAGTGAGGCACCTATCT
                                                                3060
CATATATACTCATTTGAACCAGACTGTCAATGGTTACGAATTAGTCACTCCGTGGATAGA
                                   285                 280
                              *  W  H  K  I  L  S  A  G  I  E
                                        AmpR
```

```
CAGCGATCTGTCTATTTCGTTCATCCATAGTTGCCTGACTCCCCGTCGTGTAGATAACTA
                                                                3120
GTCGCTAGACAGATAAAGCAAGTAGGTATCAACGGACTGAGGGGCAGCACATCTATTGAT
     275            270            265            260
  A  I  Q  R  N  R  E  D  M  T  A  Q  S  G  T  T  Y  I  V  V
                            AmpR
```

```
                                          Eco31I
CGATACGGGAGGGCTTACCATCTGGCCCCAGTGCTGCAATGATACCGCGAGACCCACGCT
                                                                3180
GCTATGCCCTCCCGAATGGTAGACCGGGGTCACGACGTTACTATGGCGCTCTGGGTGCGA
     255            250            245            240
  I  R  S  P  K  G  D  P  G  L  A  A  I  I  G  R  S  G  R  E
                            AmpR
```

```
CACCGGCTCCAGATTTATCAGCAATAAACCAGCCAGCCGGAAGGGCCGAGCGCAGAAGTG
                                                                3240
GTGGCCGAGGTCTAAATAGTCGTTATTTGGTCGGTCGGCCTTCCCGGCTCGCGTCTTCAC
     235            230            225            220
  G  A  G  S  K  D  A  I  F  W  G  A  P  L  A  S  R  L  L  P
                            AmpR
```

```
    EciI                       VspI
GTCCTGCAACTTTATCCGCCTCCATCCAGTCTATTAATTGTTGCCGGGAAGCTAGAGTAA
                                                                3300
CAGGACGTTGAAATAGGCGGAGGTAGGTCAGATAATTAACAACGGCCCTTCGATCTCATT
     215            210            205            200
  G  A  V  K  D  A  E  M  W  D  I  L  Q  Q  R  S  A  L  T  L
                            AmpR
```

```
GTAGTTCGCCAGTTAATAGTTTGCGCAACGTTGTTGCCATTGCTGCAGGCATCGTGGTGT
                                                                3360
CATCAAGCGGTCAATTATCAAACGCGTTGCAACAACGGTAACGACGTCCGTAGCACCACA
     195            190            185            180
  L  E  G  T  L  L  K  R  L  T  T  A  M  A  A  P  M  T  T  D
                            AmpR
```

CACGCTCGTCGTTTGGTATGGCTTCATTCAGCTCCGGTTCCCAACGATCAAGGCGAGTTA 3420
GTGCGAGCAGCAAACCATACCGAAGTAAGTCGAGGCCAAGGGTTGCTAGTTCCGCTCAAT

175 170 165 160
R E D N P I A E N L E P E W R D L R T V
AmpR

CATGATCCCCCATGTTGTGCAAAAAAGCGGTTAGCTCCTTCGGTCCTCCGATCGTTGTCA 3480
GTACTAGGGGGTACAACACGTTTTTTCGCCAATCGAGGAAGCCAGGAGGCTAGCAACAGT

155 150 145 140
H D G M N H L F A T L E K P G G I T T L
AmpR

DnaA box6

GAAGTAAGTTGGCCGCAGTGTTATCACTCATGGTTATGGCAGCACTGCATAATTCTCTTA 3540
CTTCATTCAACCGGCGTCACAATAGTGAGTACCAATACCGTCGTGACGTATTAAGAGAAT

135 130 125 120
L L N A A T N D S M T I A A S C L E R V
AmpR

CTGTCATGCCATCCGTAAGATGCTTTTCTGTGACTGGTGAGTACTCAACCAAGTCATTCT 3600
GACAGTACGGTAGGCATTCTACGAAAAGACACTGACCACTCATGAGTTGGTTCAGTAAGA

115 110 105 100
T M G D T L H K E T V P S Y E V L D N Q
AmpR

GAGAATAGTGTATGCGGCGACCGAGTTGCTCTTGCCCGGCGTCAACACGGGATAATACCG 3660
CTCTTATCACATACGCCGCTGGCTCAACGAGAACGGGCCGCAGTTGTGCCCTATTATGGC

95 90 85 80
S Y H I R R G L Q E Q G A D V R S L V A
AmpR

CGCCACATAGCAGAACTTTAAAAGTGCTCATCATTGGAAAACGTTCTTCGGGGCGAAAAC 3720
GCGGTGTATCGTCTTGAAATTTTCACGAGTAGTAACCTTTTGCAAGAAGCCCCGCTTTTG

75 70 65 60
G C L L V K F T S M M P F R E E P R F S
AmpR

TCTCAAGGATCTTACCGCTGTTGAGATCCAGTTCGATGTAACCCACTCGTGCACCCAACT 3780
AGAGTTCCTAGAATGGCGACAACTCTAGGTCAAGCTACATTGGGTGAGCACGTGGGTTGA

55 50 45 40
E L I K G S N L D L E I Y G V R A G L Q
AmpR

GATCTTCAGCATCTTTTACTTTCACCAGCGTTTCTGGGTGAGCAAAAACAGGAAGGCAAA 3840
CTAGAAGTCGTAGAAAATGAAAGTGGTCGCAAAGACCCACTCGTTTTTGTCCTTCCGTTT

35 30 25 20
D E A D K V K V L T E P H A F V P L C F
signal sequence
AmpR

ATGCCGCAAAAAAGGGAATAAGGGCGACACGGAAATGTTGAATACTCATACTCTTCCTTT 3900
TACGGCGTTTTTTCCCTTATTCCCGCTGTGCCTTTACAACTTATGAGTATGAGAAGGAAA

15 10 5 1
A A F F P I L A V R F H Q I S M
signal sequence
AmpR
AmpR promoter

TTCAATATTATTGAAGCATTTATCAGGGTTATTGTCTCATGAGCGGATACATATTTGAAT 3960
AAGTTATAATAACTTCGTAAATAGTCCCAATAACAGAGTACTCGCCTATGTATAAACTTA
AmpR promoter

GTATTTAGAAAAATAAACAAATAGGGGTTCCGCGCACATTTCCCCGAAAAGTGCCACCTG 4020
CATAAATCTTTTTATTTGTTTATCCCCAAGGCGCGTGTAAAGGGGCTTTTCACGGTGGAC
AmpR promoter

AatII
ACGTCTAAGAAACCATTATTATCATGACATTAACCTATAAAAATAGGCGTATCACGAGGC 4080
TGCAGATTCTTTGGTAATAATAGTACTGTAATTGGATATTTTTATCCGCATAGTGCTCCG

AanI
CCTTTCGTCTTCAAGAATTTTATAAACCGTGGAGCGGGCAATACTGAGCTGATGAGCAAT 4140
GGAAAGCAGAAGTTCTTAAAATATTTGGCACCTCGCCCGTTATGACTCGACTACTCGTTA

TTCCGTTGCACCAGTGCCCTTCTGATGAAGCGTCAGCACGACGTTCCTGTCCACGGTACG 4200
AAGGCAACGTGGTCACGGGAAGACTACTTCGCAGTCGTGCTGCAAGGACAGGTGCCATGC

CCTGCGGCCAAATTTGATTCCTTTCAGCTTTGCTTCCTGTCGGCCCTCATTCGTGCGCTC 4260
GGACGCCGGTTTAAACTAAGGAAAGTCGAAACGAAGGACAGCCGGGAGTAAGCACGCGAG

BamHI
TAGGATCCACAGGACGGGTGTGGTCGCCATGATCGCGTAGTCGATAGTGGCTCCAAGTAG 4320
ATCCTAGGTGTCCTGCCCACACCAGCGGTACTAGCGCATCAGCTATCACCGAGGTTCATC
DnaA box7

CGAAGCGAGCAGGACTGGGCGGCGGCCAAAGCGGTCGGACAGTGCTCCGAGAACGGGTGC 4380
GCTTCGCTCGTCCTGACCCGCCGCCGGTTTCGCCAGCCTGTCACGAGGCTCTTGCCCACG

Eco47III

GCATAGAAATTGCATCAACGCATATAGCGCTAGCAGCACGCCATAGTGACTGGCGATGCT 4440
CGTATCTTTAACGTAGTTGCGTATATCGCGATCGTCGTGCGGTATCACTGACCGCTACGA

GTCGGAATGGACGATATCCCGCAAGAGGCCCGGCAGTACCGGCATAACCAAGCCTATGCC 4500
CAGCCTTACCTGCTATAGGGCGTTCTCCGGGCCGTCATGGCCGTATTGGTTCGGATACGG

TACAGCATCCAGGGTGACGGTGCCGAGGATGACGATGAGCGCATTGTTAGATTTCATACA 4560
ATGTCGTAGGTCCCACTGCCACGGCTCCTACTGCTACTCGCGTAACAATCTAAAGTATGT

HindIII

CGGTGCCTGACTGCGTTAGCAATTTAACTGTGATAAACTACCGCATTAAAGCTTATCGAT 4620
GCCACGGACTGACGCAATCGTTAAATTGACACTATTTGATGGCGTAATTTCGAATAGCTA

AATTTTCAATGACTGCTCAATGCCCGGATCTTCGTCACAATTCTCAAGTCGCTGATTTCA 4680
TTAAAAGTTACTGACGAGTTACGGGCCTAGAAGCAGTGTTAAGAGTTCAGCGACTAAAGT

AAAAACTGTAGTATCCTCTGCGAAACGATCCCTGTTTGAGTATTGAGGAGGCGAGATGTC 4740
TTTTTGACATCATAGGAGACGCTTTGCTAGGGACAAACTCATAACTCCTCCGCTCTACAG

GCAGACAGAAAATGCAGTGACTTCCTCATTGAGTCAAAAGCGGTTTGTGCGCAGAGGTAA 4800
CGTCTGTCTTTTACGTCACTGAAGGAGTAACTCAGTTTTCGCCAAACACGCGTCTCCATT

GCCTATGACTGACTCTGAGAAACAAATGGCCGCTGTTGCAAGAAAACGTCTTACACACAA 4860
CGGATACTGACTGAGACTCTTTGTTTACCGGCGACAACGTTCTTTTGCAGAATGTGTGTT

DnaA box 8

BglII

AGAGATAAAAGTTTTTGTCAAAAATCCTCTGAAAGATCTCATGGTTGAGTACTGCGAGAG 4920
TCTCTATTTTCAAAAACAGTTTTTAGGAGACTTTCTAGAGTACCAACTCATGACGCTCTC

AGAGGGGATAACACAGGCTCAGTTCGTTGAGAAAATCATCAAAGATGAACTGCAGAGACT 4980
TCTCCCCTATTGTGTCCGAGTCAAGCAACTCTTTTAGTAGTTTCTACTTGACGTCTCTGA

GGATATACTAAAGTAAAGACTTTACTTTGTGGCGTAGCATGCTAGATTACTGATCGTTTA 5040
CCTATATGATTTCATTTCTGAAATGAAACACCGCATCGTACGATCTAATGACTAGCAAAT

AGGAATTTTGTGGCTGGCCACGCCGTAAGGTGGCAAGGAACTGGTTCTGATGTGGATTTA 5100
TCCTTAAAACACCGACCGGTGCGGCATTCCACCGTTCCTTGACCAAGACTACACCTAAAT

```
CAGGAGCCAGAAAAGCAAAAACCCCGATAATCTTCTTCAACTTTGGCGAGTACGAAAAGA   5160
GTCCTCGGTCTTTTCGTTTTTGGGGCTATTAGAAGAAGTTGAAACCGCTCATGCTTTTCT
copA

TTACCGGGGCCCACTTAAACCGTATAGCCAACAATTCAGCTATGCGGGGAGTATAGTTAT   5220
AATGGCCCCGGGTGAATTTGGCATATCGGTTGTTAAGTCGATACGCCCCTCATATCAATA
copA
tap

ATGCCCGGAAAAGTTCAAGACTTCTTTCTGTGCTCGCTCCTTCTGCGCATTGTAAGTGCA   5280
TACGGGCCTTTTCAAGTTCTGAAGAAAGACACGAGCGAGGAAGACGCGTAACATTCACGT
tap

GGATGGTGTGACTGATCTTCACCAAACGTATTACCGCCAGGTAAAGAACCCGAATCCGGT   5340
CCTACCACACTGACTAGAAGTGGTTTGCATAATGGCGGTCCATTTCTTGGGCTTAGGCCA
tap

GTTCACTCCCCGTGAAGGTGCCGGAACGCTGAAGTTCTGCGAAAAACTGATGGAAAAGGC   5400
CAAGTGAGGGGCACTTCCACGGCCTTGCGACTTCAAGACGCTTTTTGACTACCTTTTCCG
ORF1

GGTGGGCTTCACCTCCCGTTTTGATTTCGCCATTCATGTGGCGCATGCCCGTTCCCGTGG   5460
CCACCCGAAGTGGAGGGCAAAACTAAAGCGGTAAGTACACCGCGTACGGGCAAGGGCACC
ORF1

SalI
SgrDI
TCTGCGTCGGCGCATGCCACCGGTGCTGCGTCGACGGGCTATTGATGCGCTGCTGCAGGG   5520
AGACGCAGCCGCGTACGGTGGCCACGACGCAGCTGCCCGATAACTACGCGACGACGTCCC
ORF1

GCTGTGTTTCCACTATGACCCGCTGGCCAACCGCGTCCAGTGTTCCATCACCACACTGGC   5580
CGACACAAAGGTGATACTGGGCGACCGGTTGGCGCAGGTCACAAGGTAGTGGTGTGACCG
ORF1

CATTGAGTGCGGACTGGCGACAGAGTCCGGTGCAGGAAAACTCTCCATCACCCGTGCCAC   5640
GTAACTCACGCCTGACCGCTGTCTCAGGCCACGTCCTTTTGAGAGGTAGTGGGCACGGTG
ORF1

SmaI
CCGGGCCCTGACGTTCCTGTCAGAGCTGGGACTGATTACCTACCAGACGGAATATGACCC   5700
GGCCCGGGACTGCAAGGACAGTCTCGACCCTGACTAATGGATGGTCTGCCTTATACTGGG
ORF1
```

```
GCTTATCGGGTGCTACATTCCGACCGACATCACGTTCACACTGGCTCTGTTTGCTGCCCT   5760
CGAATAGCCCACGATGTAAGGCTGGCTGTAGTGCAAGTGTGACCGAGACAAACGACGGGA
ORF1 >

                                           PauI
TGATGTGTCTGAGGATGCAGTGGCAGCTGCGCGCCGCAGTCGTGTTGAATGGGAAAACAA   5820
ACTACACAGACTCCTACGTCACCGTCGACGCGCGGCGTCAGCACAACTTACCCTTTTGTT
ORF1 >

                                          PasI
ACAGCGCAAAAAGCAGGGGCTGGATACCCTGGGTATGGATGAGCTGATAGCGAAAGCCTG   5880
TGTCGCGTTTTTCGTCCCCGACCTATGGGACCCATACCTACTCGACTATCGCTTTCGGAC
ORF1 >

                                          BspTI
GCGTTTTGTGCGTGAGCGTTTCCGCAGTTACCAGACAGAGCTTAAGTCCCGTGGAATAAA   5940
CGCAAAACACGCACTCGCAAAGGCGTCAATGGTCTGTCTCGAATTCAGGGCACCTTATTT
ORF1 >

ACGTGCCCGTGCGCGTCGTGATGCGAACAGAGAACGTCAGGATATCGTCACCCTGGTGAA   6000
TGCACGGGCACGCGCAGCACTACGCTTGTCTCTTGCAGTCCTATAGCAGTGGGACCACTT
ORF1 >

ACGGCAGCTGACGCGTGAAATCTCGGAAGGACGCTTCACTGCTAATGGTGAGGCGGTAAA   6060
TGCCGTCGACTGCGCACTTTAGAGCCTTCCTGCGAAGTGACGATTACCACTCCGCCATTT
ORF1 >

ACGCGAAGTGGAGCGTCGTGTGAAGGAGCGCATGATTCTGTCACGTAACCGCAATTACAG   6120
TGCGCTTCACCTCGCAGCACACTTCCTCGCGTACTAAGACAGTGCATTGGCGTTAATGTC
ORF1 >

CCGGCTGGCCACAGCTTCTCCCTGAAAGTGATCTCCTCAGAATAATCCGGCCTGCGCCGG   6180
GGCCGACCGGTGTCGAAGAGGGACTTTCACTAGAGGAGTCTTATTAGGCCGGACGCGGCC
ORF1 >  RepA >

AGGCATCCGCACGCCTGAAGCCCGCCGGTGCACAAAAAAACAGCGTCGCATGCAAAAAAC   6240
TCCGTAGGCGTGCGGACTTCGGGCGGCCACGTGTTTTTTTGTCGCAGCGTACGTTTTTTG
RepA >

AATCTCATCATCCACCTTCTGGAGCATCCGATTCCCCCTGTTTTTAATACAAAATACGCC   6300
TTAGAGTAGTAGGTGGAAGACCTCGTAGGCTAAGGGGGACAAAAATTATGTTTTATGCGG
RepA >
```

BbvCI
Bpu10I

```
TCAGCGACGGGGAATT     3′
                          6316
AGTCGCTGCCCCTTAA     5′
```

RepA
